# Tribute of Respect

**Published:** 1872-05

**Authors:** G. W. Priest, Wm. H. Goddard

**Affiliations:** Secretary; Chairman


### Tribute of Respect.

At a meeting of the dental profession of the city of Louisville, Ky., to take into consideration such action as to them seemed appropriate on the occasion of the death of Dr. E. W. Mason, dentist of this city, on motion of Dr. J. F. Canine, Dr. W. H. Goddard was called to the chair, and Dr. Priest elected Secretary. After several handsome tributes of respect and eulogies upon the life and character of the deceased by the chairman and others, Drs. Priest, Redman and Dunn were appointed a committee to draft such resolutions as would be appropriate to the occasion. The following were unanimously adopted:

WHEREAS, We have heard with deep regret of the sudden death of our professional brother and co-laborer, Dr. E. W. Mason, and, desirous of expressing our high appreciation of his pre-eminent skill and ability, his earnest endeavor to elevate and maintain the profession in the highest perfection of the art; therefore,

*Resolved*, That in the death of Dr. Mason the profession has lost one of its most honored and worthy members; one who, until stricken down by disease, devoted his whole life assiduously to his calling, Courteous and kind to his professional brethren, industrious and zealous in the prosecution of his duties, a friend of the poor and distressed, with a heart always ready to respond to the demands of the afflicted, his death will be deeply felt and regretted by this community, in which he lived.

*Resolved*, That we tender the stricken family and friends our heartfelt condolence and sympathy, assuring them that through the evidences of his life what has proven their loss is his eternal gain.

*Resolved*, That the profession attend the funeral in a body.

*Resolved*, That the newspapers of our city, together with the dental journals of the country, be requested to publish these resolutions, and a copy of the same be handed the family of the deceased by the Secretary.

WM. H. GODDARD, Chairman.
G. W. PRIEST, Secretary.

